# Supplementary material for: Are carbon emissions trading and green financial instruments synergistic? -Comprehensive quantitative research based on content analysis
Source: PLoS One. 2024 Mar 7;19(3):e0298601. doi: 10.1371/journal.pone.0298601 (PMC10919601; doi:10.1371/journal.pone.0298601)
Supplement: S1 Data — (ZIP) [file pone.0298601.s001.zip › Data/地级市Z_F工作报告.zip!/╝¬┴╓/░╫╔╜╩╨/└┤╫╘╣1⁄2╓┌║┼í╛┬φ┐╦╩2╛▌═°í┐_2016.docx]

各位代表：

　　现在，我代表市政府向大会报告工作，请予审议，并请列席人员提出意见。

　　一、回顾“十二五”，白山转型振兴迈出坚实步伐

　　2011年以来，我们在省委、省政府和市委的坚强领导下，在市人大、市政协的监督支持下，紧紧抓住振兴东北老工业基地和资源型城市经济转型试点等战略机遇，牢牢把握稳中求进总基调，积极应对经济下行和结构调整双重挑战，团结带领全市各族人民，务实创新、拼搏苦干，基本完成了“十二五”目标任务。

　　——过去五年，克难求进稳增长，经济实力稳步提升

　　经过全市上下共同努力，经济保持持续稳定健康发展。经济总量实现突破。预计2015年，实现地区生产总值710亿元，是2010年的1.6倍，年均增长9.7%，其中一、二、三产业增加值61.1亿元、414.6亿元、234.3亿元，年均增长5%、10.9%、8.5%。人均GDP达到5.6万元。财政收支实现突破。完成全口径财政收入63亿元、地方级财政收入45亿元，年均增长8%和12%；财政支出140亿元，年均增长7.3%。固定资产投资实现突破。完成固投641亿元，年均增长21.7%。累计完成2706亿元，是前五年的1.7倍。实施3000万元以上项目1800个，其中亿元以上项目680个。

　　——过去五年，提质增效调结构，转型升级扎实推进

　　全力实施产业集群梯次推进计划，多点支撑、多元发展格局初步形成。三次产业比重由2010年的10.3:60.1:29.6调整到8.8:58.2:33。传统产业优化整合。加快以通化矿业为主体的煤炭行业战略重组，72家煤矿整合为21户煤业公司，企业实现生产规模化和安全标准化。林业产品远销欧美市场，成为我市外贸进出口中坚力量，金隆木业出口创汇居全国同行业首位，三林木业打造全国规模最大、品类最全木业行业O2O电商平台正式上线。接续产业发展壮大。我市被列为全省唯一国家级整装勘查区，新增资源储量潜在价值超万亿元，矿产新材料有望成为首个千亿级支柱产业。恒大冰泉打响全球营销战略，鹿鸣甘露获全球首个清真优质认证，农夫山泉、天士力引领高端产品，泉阳泉等本土品牌迅速崛起，矿泉城国内闻名、走向世界。人参国家地理标志保护产品实现广覆盖，万良人参市场被列为省部共建国家重点专业市场，开发药食同源产品104个，“四大园区、四大基地、五大中心”初具规模。长白山国际度假区龙头作用凸显，开启全境游、四季游新时代，白山荣膺“最美中国?绿色生态、休闲度假健康（养生）旅游目的地城市”称号。预计2015年接待国内外游客750万人次，实现总收入95亿元，是2010年的2.1倍和3.1倍，年均增长15%和25%。左旋氨氯地平系列产品成为民族降压药第一品牌，长白山药业投产，葵花药业落户白山，医药产业产值翻番。接续产业增加值占GDP比重由35%提高到60%。现代服务业稳步提升。成功引进欧亚、广泽等一批城市商业综合体，完成万村千乡市场工程。智慧城市和长白山云计算中心启动，我市成为全省唯一宽带中国示范市，江源、临江列为国家智慧城市试点。长白山农特产品电商平台搭建，临江被列为全国电商进农村示范县，抚松被列为省级电商基地。预计2015年实现社会消费品零售总额266亿元，是2010年的1.9倍，年均增长13.8%。创新能力持续增强。健全政产学研用协作机制，新增2个院士工作站，国家镁合金产业化基地和全省镁产业、硅藻土产业技术创新联盟落户白山。获批省级“双十”项目7项、科学技术奖15项，投产新产品80个。国家高新技术和省创新型科技企业达到6户和11户。施慧达、喜丰被评为国家知识产权优势企业，获中国专利优秀奖。

　　——过去五年，建管并重优环境，城乡面貌持续改观

　　坚持规划引领、强化基础、功能配套，区域环境明显改善。基础设施进一步完善。完成交通建设投资224.3亿元，全市公路、铁路总里程达到6546公里和440公里。鹤大及辉临高速、四白铁路稳步推进，宇松铁路建成，营松高速及三道沟至集安界、东岗至长白、大栗子至错草等干线公路竣工通车。新改建各类管网241.8公里，地下综合管廊建设启动。加固城市堤防120公里，完成25座小型水库建设和除险加固。城乡环境进一步提升。大力实施街巷路改扩建、市区垃圾处理场和净水厂扩建、管道燃气和供水管网改造、集中供热三期、城市内河治理等52项工程，市区人均绿地面积达到26.1平方米。靖宇、江源、抚松等中心城镇建设有序推进，抚松、临江跻身“中国百佳深呼吸小城”。建成60个高标准示范村、60个整体推进村、257个“四清四改”村，村容村貌大为改观，试点村安全饮水率、通电率达到100%。生态环境进一步优化。深入推进天保工程和“三江”流域综合治理，增加森林面积2.2万公顷、林木蓄积量451万立方米。珠宝河、五道沟湿地公园和原麝保护区晋升国家级，靖宇白浆泉被评为“中国最佳水源地”。城镇生活污水和垃圾无害化处理率达80%以上，单位GDP能耗下降16.3%，超额完成“十二五”减排目标。我市被评为中国生态文明价值城市。

　　——过去五年，深化改革促开放，发展活力不断增强

　　坚持激活内力与借助外力相结合，围绕破解发展难题推改革，立足提升发展速度促开放。重点领域改革有序推进。市县两级政府机构改革完成。落实户籍和商事制度改革。全面推进县级公立医院综合改革，所有药品实现网上采购和零差率销售。林地确权基本完成，农村土地确权登记发证开展试点。获批国家主体功能区示范试点，建立绿色转型考评指标体系。对外开放水平不断提高。东北东部区域合作上升为国家战略，长白国家重点开发开放试验区纳入振兴东北规划，白山逐步融入“一带一路”和新一轮东北振兴大格局。累计引进市外资金2128亿元，实际利用外资10.5亿美元，是前五年的2.9倍和2.4倍。进出口经营权企业增至400户，出口产品达到165种。实现外贸进出口总额15.1亿美元，是前五年的1.6倍。民营经济得到长足发展。累计出台91条鼓励民营经济发展的政策措施，为企业减免税费52.6亿元。市中小企业公共服务网络平台实现视频会议、在线培训、在线申报、技术难题寻解集中服务。中小企业创业孵化基地达到10个，在孵企业139户。喜丰节水器材成功上市。个体工商户和民企户数达到6.6万户和9000户，是2010年的1.4倍和2倍。民营经济增加值占GDP比重达到54.2%。

　　——过去五年，改善民生促和谐，发展更加惠民利民

　　始终保持新增财力80%以上用于民生，累计实施民生实事157件。城乡居民人均可支配收入达到28000元和9288元，是2010年的1.7倍和1.5倍，年均增长11.4%和8.7%。居民储蓄存款余额420亿元，人均3.34万元。保障体系不断健全。累计新增城镇就业19.5万人，城镇登记失业率控制在3.9%。五大保险征缴扩面，为城乡困难群体发放低保等各类救助金17.7亿元。新农合参合率99.8%。保障性安居工程完成941.7万平方米。“暖房子”工程被列为国家级重点示范市，改造8万户477万平方米。社会事业全面发展。教育均衡水平逐年提高，白山职业技术学校被列为第三批国家中等职业教育改革发展示范校。完成68家卫生预防接种门诊规范化和441所村卫生室标准化建设，新增医疗机构201家。新改扩建文化基础设施 500余个，市博物馆、长白山美术馆、松花石博物馆纳入国家免费开放名录，公共文化服务配送被列为国家示范项目。城乡健身设施覆盖率达到78%和63%，获得全国群众体育先进单位和全民健身活动优秀组织奖。两次被评为全国双拥模范城。全市宽带覆盖率和光纤化改造率均达到99%。社会保持和谐稳定。平安白山建设成效显著，人民群众安全感满意度连续5年居全省前列。建成边境、城市、农村、国省干线“四张监控网”。全面实现安全生产“五级五覆盖”和“五落实五到位”。健全涉法涉诉信访导入司法机制，化解重大信访积案189件。全面夺取2013年抗洪抢险和灾后重建胜利。实现连续36年无重大森林火灾。

　　——过去五年，转变作风提效能，政府自身建设成效明显

　　大力推行“放管服”三结合，完成权力清单任务，启动责任清单建设，市本级审批事项由246项减少到201项，清理规范性文件44件。完善科学民主决策、政务公开、督查落实等各项政府工作规则。在全省率先实行行政 “大复议”试点。深入推进群众路线教育实践活动和“三严三实”专题教育，落实162项整改和制度建设任务，查处政府系统违反八项规定问题80余起。实施权力入笼工程，开展政行风电视听政。办理人大代表建议303件、政协委员提案347件，面复率和满意率100%。

　　五年来，统计、粮食、供销、水文、气象、保险、人防、电力、烟草、石油、盐务、民兵预备役、外事、地方志、档案、老龄、妇女儿童、红十字会等工作都为全市经济社会发展作出了积极的贡献。

　　各位代表！过去的五年，面对新世纪以来最严峻的挑战，我们团结拼搏、砥砺前行，走过的历程充满艰辛，取得的成绩令人鼓舞。我们始终坚持发展为要、加快转型，把稳增长调结构作为破解一切问题的关键，积聚振兴崛起能量；始终坚持改革为先、扩大开放，努力破除机制体制障碍，增强经济发展后劲；始终坚持统筹为策、协调推进，五位一体全面进步；始终坚持民生为本、利民惠民，让全市人民更多更好享受改革发展成果；始终坚持团结为重、凝心聚力，形成干事创业、共谋发展的浓厚氛围。这些宝贵的财富已经转化为推动白山发展的强大动力。

　　今日白山，已站在一个新的历史起点，成绩来之不易，发展催人奋进。这是省委、省政府和市委坚强领导的结果，是市人大、市政协和社会各界监督支持的结果，是全市人民拼搏奋进的结果。在此，我代表市政府向全市各族人民，向驻白山人民解放军指战员、武警部队官兵、公安司法干警、中省直单位，向各民主党派、工商联、人民团体、无党派人士、离退休老同志，向关心支持白山发展的各界朋友，致以崇高的敬意和衷心的感谢！

　　在看到成绩的同时，我们也清醒地认识到前进中存在的突出问题，主要表现在：经济总量不大，产业结构不优，转型速度不快，创新能力不强，服务水平不高，城乡环境、民生保障还需进一步提升等。对此，我们将采取有力措施，认真加以解决。

　　二、展望“十三五”，全力打造国家绿色转型发展示范区

　　未来五年，我市将进入转方式调结构的关键期、建机制增活力的攻坚期、新优势新动力的形成期、全面建成小康的决战期。放眼全球，绿色经济正成为新趋势和新潮流；面向国内，绿色发展理念和实践已贯穿到五位一体总体布局之中；立足吉林，国家实施新一轮东北振兴，省委、省政府正在加快建设东部绿色转型发展区。这些都为我们加快绿色转型、实现绿色崛起提供了良好条件，注入了强劲动力。面对诸多矛盾叠加、机遇挑战并存，我们将以高度的历史责任感和时代紧迫感，主动适应经济新常态，科学把握发展新规律，牢固树立绿色发展理念，加快供给侧结构性改革，从根本上实现经济增长动力转换和能级跃升，走出一条质量更高、效益更好、结构更优、优势充分释放的新路。

　　根据市委六届七次全会部署，今后五年政府工作的总体要求是：深入贯彻党的十八大和十八届三中、四中、五中全会精神，认真落实习近平总书记系列重要讲话精神，准确把握“四个全面”、“五大发展理念”、“五大任务”和省委、省政府“三个五”战略部署，突出生态和民生两个关键，坚持生态立市、产业强市、特色兴市, 以绿色转型为主线，以改革创新为动力，切实转变经济发展方式，加强资源能源节约和生态环境保护，繁荣发展社会各项事业，不断提高人民生活水平，建设吉林东部绿色转型先行区，全力打造国家绿色转型发展示范区。

　　经济发展主要目标是实现“七个翻番”：到2020年，全市GDP实现980亿元，年均增长6.7%；地方级财政收入60亿元，年均增长6%；规上工业增加值490亿元，年均增长6.5%；固定资产投资1032亿元，年均增长10%；社会消费品零售总额410亿元，年均增长9%；城乡居民人均可支配收入与经济增长同步。全部实现比2010年翻番目标。

　　（一）产业发展要有新突破，倾力打造实力白山

　　坚定不移推进存量经济绿色化改造和增量经济绿色化构建，形成以绿色、低碳、循环为特征的白山经济“升级版”。三次产业比重调整到7:53:40。

　　——突出发展五大产业。以创新创造为动力、结构优化为目标、项目建设为抓手，着力构建生态产业体系，打造绿色转型第一引擎。矿产新材料产业：全力推进找矿新突破，大力开发高科技含量、高附加值产品。到2020年，形成硅、镁、硅藻土等产业链条，实现产值800亿元，打造亚洲硅藻土之都、中国镁谷和东北重要矿产新材料基地。矿泉水产业：坚持保护为先、集约利用，开发中高端矿泉饮品，加大物流运输调配力度，加快发展围水包装产业。到2020年，天然矿泉饮品产能达到3000万吨，实现产值300亿元，打造国际矿泉城。医药健康产业：坚持以人参为龙头，壮大中药材GAP种植基地，统筹发展药品、保健品、绿色食品以及健康养老业。到2020年，实现产值600亿元，打造中国人参城、长白山北药生产基地和休闲养生之都。旅游产业：发挥长白山国际度假区龙头作用，开发精品线路，完善配套设施，形成“依山、沿江、环城、跨国”新格局。到2020年，接待国内外游客突破1500万人次，实现旅游总收入300亿元，打造国际旅游休闲度假目的地，创建中国优秀旅游城。现代服务业：突出发展管理咨询、现代物流、信息技术等生产性服务业和社区、商贸、家政等生活性服务业。统筹发展服务外包、电子商务等新兴业态，着力培育百亿级电商平台。到2020年，实现产值850亿元，打造长白山区域物流枢纽和现代服务业中心。

　　——全面实施“十百万千”计划。坚持园区带动，统筹规划布局，推进各类要素集聚，实现资源集约、产业集群发展，把园区建设成为引领转型的先导区和壮大市区经济、推动县域加快发展的重要载体。到2020年园区经济占全市总量70%以上，建成产值50亿元特色园区10个；坚持民营经济推动，优化市场主体发展环境，加大政策扶持力度，营造大众创业、万众创新浓厚氛围，打造一批“双创”基地，争创全国中小企业发展环境示范市，新增 “四上”企业200户，民企达到14500户；坚持创新和品牌驱动，加快创新体系建设，推进标准化管理，打造一批企业技术创新中心、科技成果交易中心、公共研发平台和院士工作站，提升自主创新能力，推进科技成果转化，培育高新技术企业，争创全省知名品牌示范区，研发新产品1000个。

　　（二）城市建设要有新形象，倾力打造魅力白山

　　坚持统筹做好规划建设管理，优化空间、完善功能、打造精品、彰显特色，创建国家园林城、卫生城、文明城，构建宜居宜业佳地。

　　——推进市区一体化。推动浑江、江源两区东联西拓、南控北跨、组团发展，构建“五横九纵”城市街路框架和“一大环、五小环、多支线”地下综合管廊体系，建设城市备用水源。以城市管理现代化为指向，加快城市规划、产业布局、园区建设、基础设施、体制机制、公共服务“六个一体化”，打造市区协同发展、共享共建格局，增强城市吸纳、辐射和带动能力，建设50万人口长白山核心区域中心城市。

　　——推进城市景观化。以创建水生态文明城市为载体，以绿为脉、以文为蕴，打造山水相依、街景相宜、山水城交融城市景观系统。推进北山公园和青山湖融合连通，改造一江十河和江堤公园，形成水清堤靓、岸绿河畅的绿色休闲景观带。建设10个文体场馆、3个样板小区、14条风情街路、12个广场公园，以及一批具有文化底蕴和长白山区域特色的景观小品，提升城市品位。

　　——推进城镇特色化。坚持区域协同、城乡一体，发展特色经济，推进以人为核心的新型城镇化建设。打造10个旅游度假、农林特产、商贸物流、工矿、边贸 “五型城镇”，建成10个美丽乡镇、200个美丽乡村、3万户美丽庭院、5万户干净人家。全市城镇化率达到70.8%，形成“双核三轴十心多点”空间格局。

　　——推进交通立体化。确保鹤大和辉白高速竣工通车，加快建设白临、松长高速，新改建沈阳至佳木斯、四平至白河、松江河至长白、靖宇至辉南等铁路以及三道沟至错草、靖宇至江源、漫江至临江等干线公路，力争新机场开工。融入陆海联运大通道，构建“六横七纵两口岸一机场”东北东部区域交通枢纽。

　　——推进服务信息化。大力推进宽带中国示范市和长白山云计算中心建设，实现移动通信3G、4G全覆盖，探索政务大数据和互联网+公共服务模式，建成行政审批和便民服务网，政府服务事项上网率90%以上，协调推进“四化”融合，全面建成智慧白山。

　　（三）改革开放要有新层次，倾力打造活力白山

　　坚持深化改革、扩大开放，积极构建全方位、宽领域、纵深化改革开放新格局，努力扩大白山经济外向度。

　　——构建开放合作体系。充分利用国际、国内两种资源、两个市场，加快建设长白国家重点开发开放试验区和长白、临江边境经济合作区，积极争设边境互市贸易区，更加主动融入“一带一路”、东北东部区域合作和白通丹鸭绿江经济带，促进区域间资源、信息共享流动。坚持引资、引智、引技相结合，精准有效承接产业转移，建成东北东部沿边开发开放桥头堡。

　　——构建开放金融体系。大力发展有利于中小企业成长、技术创新和产业结构调整的信贷服务机构，鼓励社会资本设立民营银行和投资公司。加快发展类金融产业，争取举办长白山金融论坛，构筑金融会展经济高地。设立政策性引导基金和种子基金，发展股权融资、风险投资、互联网金融等新业态，强力推进资产证券化和直接融资，力争全市挂牌上市企业达到30户以上，实现金融机构多元化、产品多样化、市场多层次化、业务特色化和服务精细化。

　　——构建开放市场体系。完善政策措施，消除市场壁垒，鼓励支持社会资本进入基础设施、公共服务等领域开展合作。健全土地承包经营权流转市场，稳步开展农村集体经济组织产权制度改革和集体建设地流转试点。紧跟顶层设计，确保重点领域和关键环节改革取得决定性进展，形成产权清晰、流动顺畅、统一开放、竞争有序的现代市场体系，让财富源泉充分涌流。

　　（四）生态环境要有新提升，倾力打造绿色白山

　　坚持尊重自然、顺应自然、保护自然，建设天蓝水净、地绿山青的东北亚重要生态屏障，争创国家级生态文明建设示范市。

　　——实施污染防治行动。严控大气和水污染，严格限制“三高”产能，积极发展可再生和清洁能源，推进绿色能源乡村建设，推广以电代燃。城市工业用水重复利用率达到80%以上，市区及县城空气质量达标天数均超过240天。生活和医疗垃圾等有毒有害废物处置率达到100%，生活污水处理达标率在80%以上，工业用水处理达标率在90%以上，主要江河水质达到三类水体标准，城镇饮用水源地水质100%达标。

　　——实施生态修复行动。严守生态红线，合理有序推进生态移民。抓好新一轮退耕还林、三北五期和封山育林，推进森林、湿地公园和自然保护区建设。到2020年，土地复垦和矿山复绿6000公顷，改造低质国有林15万公顷，自然保护区面积达到20万公顷，森林面积增加2.7万公顷，森林覆盖率达到84.5%，构建“两区三带多斑块”生态功能区格局。

　　——实施生态文明行动。坚持和完善最严格的耕地保护制度，盘活闲置低效利用土地，提高单位面积投资强度和产出水平。煤矸石、粉煤灰、低品位硅藻土等工业固废物综合利用率达到70%以上，单位GDP能耗降低15%。健全生态保护规划体系，探索地区间生态补偿机制，建立用能、用水、排污及碳排放权初始分配制度，积极争设国家森林碳汇交易和绿色GDP核算试点地区。

　　（五）社会事业要有新气象，倾力打造幸福白山

　　坚持发展为了人民，发展成果由人民共享，切实增强人民群众福祉感、获得感、快乐感。

　　——推进精准脱贫工程。通过发展生产脱贫一批、易地搬迁脱贫一批、生态补偿脱贫一批、发展教育脱贫一批、社会保障兜底一批，设定时间表，逐户逐人销号，打好帮扶“组合拳”，打赢脱贫攻坚战，确保贫困人口全部脱贫，靖宇摘掉国家级贫困县帽子。

　　——推进收入提升工程。大力促进就业再就业，增加工资性收入，提升居民经营性、财产性和创业收入。到2020年，累计创造稳定就业岗位5万个，城镇新增就业18万人、农村劳动力转移就业10万人，发放创业担保贷款4亿元，全面消除零就业家庭，实现低收入人群收入翻番。

　　——推进社会保障工程。坚持保基本、兜底线，实现养老、医疗、失业、工伤、生育保险政策性全覆盖，切实提高城乡低保、社会福利、大病救助和救灾救济水平。

　　——推进文教普惠工程。加快文化基础设施建设，增强公共文化产品和服务供给，塑造地域特色文化品牌，提升市民精神风貌。促进义务教育均衡发展，普及高中阶段教育，大力发展现代职业教育。逐步分类推进中等职业教育免除学杂费，家庭经济困难学生资助全覆盖。

　　——推进健康白山工程。全面深化公立医院改革，健全城乡医疗卫生服务网络，实现卫生服务进社区全覆盖。落实“全面两孩”政策，提高出生人口素质，优化性别比例。着力构建“四大”监管平台，完善“六大”工作体系，创建食品药品安全放心城市。发展竞技体育和全民健身，争创国家高水平体育后备人才基地，打造城市社区“15分钟健身圈”。

　　——推进平安白山工程。深化依法治市，健全社会立体防控体系，维护社会稳定。巩固完善四化融合、三位一体安全监管防控体系，实现标准化全达标、信息化全覆盖，确保安全发展。提升防震减灾能力，强化地质灾害防治。加强民族宗教工作，抓好边境安全管控，建成全国最平安城市和最稳定边境地区。

　　各位代表！经过未来五年的不懈努力，一个经济繁荣、活力迸发、环境优美、生态良好、社会和谐的新白山将绿色崛起，全市人民一定会生活得更加美好幸福！

　　三、着眼新起点，努力实现“十三五”良好开局

　　2016年是“十三五”开局之年，做好今年工作事关未来五年发展。主要预期目标是：GDP增长7%左右，地方级财政收入增长3%，固定资产投资增长10％，社会消费品零售总额增长9%，城乡居民人均可支配收入与经济增长同步，城镇登记失业率控制在4.5%以内，单位GDP能耗降低3.5%。

　　实现上述目标，要突出抓好六方面重点工作：

　　（一）突出投资拉动，推进项目建设

　　提高投资有效性，加快培育新的发展动能，增强持续增长动力，实施430个3000万元以上重点项目，完成投资450亿元。一是全力抓推进。坚持重大项目领导包保、挂牌督办机制，切实解决用地、资金等瓶颈问题，推进菩提金年产200万吨矿泉水、临江医药健康产业园等230个新建项目；加快舜茂10万吨高纯二氧化硅、恒大500万吨矿泉饮品等200个续建项目；确保星泰30万吨预拌干粉砂浆、太安堂人参中药产业园等60个项目竣工投产。二是全力抓招商。深入推进招商引资冬春会战，积极组织参加世界500强和台商企业进吉林活动，创新招商理念，严把准入关口，提高招商实效，协议履约率达到60%以上，全年引进市外资金638亿元，增长10%。三是全力抓谋划。紧盯国家投资走向和市场走势，谋划260个具有标志性、引领性、成长性项目，滚动谋划100个亿元以上项目，力争更多项目挤进国家和省发展盘子。

　　（二）突出产业发展，加快结构调整

　　坚持去产能、去库存、去杠杆、降成本、补短板，提高供给体系质量和效率，推动产业优化升级。一是加快发展工业。规上企业抓增效、规下企业抓壮大、双停企业抓复产，落实217户重点企业专人包保和一对一帮扶，积极争取项目达产达效奖励、直购电等优惠政策，打好工业保增长促发展攻坚战。全年新增规上工业企业10户以上，实现规上工业增加值415亿元，增长6.5%。二是稳步发展特色农业。大力推进参、蛙、菌、药、菜、牧、果、蜂八大特色板块，加快建设五味子、蓝莓等33个特色农产品基地，培育发展大山合、北佳等36户龙头企业。依托长白山特产云商城、渤商所和邮乐网等电商平台，全力打造“互联网+长白山特产”新业态。实现农产品加工销售收入253亿元，增长10%。三是积极发展服务业。完成市区服务业规划，明确区域和产业布局，促进集聚发展。重点开发松江河、长白民俗村等一批旅游线节点，全年接待国内外游客870万人次、实现总收入120亿元，增长15%和25%。稳步发展会展经济，办好长白山鸭绿江松花江文化旅游节、松花石节等活动。实现服务业增加值253亿元，增长8%。四是大力建设产业园区。突出发展市直工业，做好城市腾空土地调规，启动规划建设“一带两区十园”，以浑江、江源两区为阵地，以浑江走廊为带，建设高新技术、矿产加工、新材料、太安大健康、机械制造、中韩科技、人参生命科技、长白山绿色食品、新型建材和金河工业园等10个园区。年内完成2—3个新建园区基础设施建设，做好相关园区城乡总体规划、控详规调整，环保红线、永久性耕地确认及土地收储等前期工作。五是着力提升服务能力。积极开展引导私营企业建立现代企业制度试点，改造升级10处孵化基地。完善银企保融资对接信息平台，实行融资联席会议和定期调度制度，充分发挥中小企业“助保金池贷款”作用，破解资金瓶颈。把握IPO注册制度改革契机，积极推动中天集团、远通矿业等企业上市。

　　（三）突出精建细管，改善人居环境

　　启动“联创“工作，重点实施六大工程，加快提升城乡面貌。一是交通建设工程。加快建设辉白高速，做好三道沟至错草、漫江至临江、靖宇至江源二级公路前期工作，鹤大高速白山段、长松岭隧道全线通车。新建农村公路150公里。二是景观提升工程。改造文化广场、希望广场，浑江大街、建设街、启明路等10条街路以及悦心苑、月园等6处公共绿地，培植绿篱、地被花卉、草坪8.6万平方米。三是功能提升工程。开工浑江区南平街、江源区正岔街地下综合管廊，建设海绵城市。开工城区供水改造工程，西北岔水源地年底蓄水。完成金河大桥主体工程，改建1座市区危桥。建设2个立体停车场。推进综合交通运输枢纽前期工作。四是综合整治工程。科学界定市区管理事权，加快推进执法重心、力量下移和综合执法，开展市容环境综合整治百日会战，重点打好城市出入口、城乡结合部、小区院落等“八大”整治战役，推动城市管理向管精、管细、管长效转变。深入实施一江十河治理。开工建设生活垃圾综合处理工程，确保完成减排任务，集中式饮用水源水质达标率100%。五是安居保障工程。实施棚改攻坚战，改造河口、碱厂沟棚户区35万平方米，货币化安置5381户。气化白山正式投入运营，新增燃气用户3000户。推进市区69栋物业弃管楼整治，探索市场化模式解决物业管理难题。六是美丽乡村工程。高标准建设22个省级重点村和24个美丽示范村，达到“六通六改六建三化”标准。打造5000个美丽庭院、1万户干净人家。完成100个村综合整治，村屯主干道两侧绿化率达到95%以上，巷道两侧绿化率在80%以上。

　　（四）突出体制创新，深化改革开放

　　把改革开放作为激发活力、增添动力的根本举措，破除体制机制障碍，更多释放改革红利。一是推进重点领域改革。深化投融资体制改革，在环保、水利、市政等领域谋划40个PPP项目。深化农村综合改革和集体林权配套制度改革，加快土地承包经营权确权登记和所有权、承包权、经营权“三权”分置并行，开展林木、参地、草场、水面等领域物权融资试点。加强生态文明制度体系建设，健全国土空间开发保护、企业环境信用评价、环境承载能力监测预警、污染物排放许可和总量控制、资源有偿使用和生态补偿等各项机制。二是大力发展民营经济。全面落实新25条政策，深入开展百名县处级干部包保百户小微企业、市直部门培育市场主体和“小升规”等专项帮扶活动。加快建设电商孵化基地和众创示范园区，利用电商平台催生壮大市场主体。实现民营经济主营业务收入1950亿元，从业人员达到37.5万人，增长7%和3.5%。三是着力扩大对外开放。完善口岸服务功能，做好长白、临江口岸通道建设前期工作。积极争取对朝出口配额和政策支持，发展边境贸易。争设出口工业品质量安全示范区，新增外贸经营权企业20户。实现外贸进出口总额3.05亿美元，增长5%。

　　（五）突出改善民生，增进群众福祉

　　进一步加大投入、兜住底线、补齐短板，办好26件民生实事，打响脱贫攻坚战。一是促进就业富民。开展就业援助，帮扶困难群体就业再就业，城镇新增就业4.2万人，农村劳动力转移就业2万人。实施“万名创业者、万名小老板”培训计划，力争年内新增小微企业1000户、个体工商户3300户。二是促进社保利民。加大征缴扩面力度，养老、医疗、失业、工伤、生育保险参保人数达到21.1万人、96.8万人、11.5万人、29.5万人、23.5万人，社保“一卡通”覆盖率达到53%，城乡低保标准达到上年度人均收入的20%和30%。三是促进医疗益民。全面深化县级公立医院改革，破除以药补医机制。建立分级诊疗制度，提高基层医疗机构服务能力。新农合参合率保持99%以上，重点对象医疗大病救助比例不低于70%，出生性别比控制在110以内。四是促进教育兴民。推动义务教育“大学区”建设以及普通高中多样化、特色化发展，加快发展学前教育，新改建11所公办幼儿园，扶持98所普惠性幼儿园。继续实施农村特岗教师计划，扩大公办校教师合理交流轮岗，促进教育均衡发展。进一步健全家庭经济困难学生资助体系。加快推进长白山职业技术学院异地新建工程。五是促进文体惠民。积极创建国家公共文化服务体系示范区，推进四馆免费开放，组织开展“两节”等各类群众性文化活动。建成92个村庄社区和8个乡镇街道全民健身设施以及3个笼式足球场、1个多功能健身中心、1个健身广场、1个户外活动基地。六是促进和谐安民。开展平安社区、乡镇、校园、企业、集市“五安工程”，严查公共安全隐患，严打各类犯罪，提高群众安全感。强化党政军警民企“六位一体”综合治边，协调周边地区建立泛鸭绿江区域警务协作机制，保障边境安全。推行“阳光信访”新模式，积极回应群众诉求。毫不松懈地开展安全生产大检查大整改和专项行动，坚决遏制各类事故。深入开展餐饮服务食品安全示范县、示范街、示范店建设，实施药品医疗器械“黑名单”制度，确保群众饮食用药安全。实现连续37年无重大森林火灾。

　　（六）突出职能转变，加强政府自身建设

　　以严的要求、实的作风，持续把转变政府职能推向深入，着力打造务实高效、法治廉洁政府。一是放权简政。坚持“放管服”三管齐下，承接落实好上级下放取消的审批事项，力争再削减一批行政审批和核准项目、一批审批中介事项、一批企业登记注册和办事关卡，实现重大项目“并联审批”，加快建立“四单一网”，变多头受理为一口受理，进一步优化流程、提高服务效率和质量。二是依法行政。完善重大事项科学民主决策制度。推动机构、职能、权限、程序、责任法定化，切实做到法无授权不可为、法定职责必须为。建立健全“双随机”抽查、行政裁量权基准、行政执法责任制和重大行政执法决定法制审核等各项制度，严格规范公正文明执法。自觉接受人大、政协、司法、社会和舆论监督，认真办理人大代表建议和政协委员提案。三是廉洁从政。全面落实“两个责任”和“一岗双责”，切实负起廉政建设主体责任，加强对政府班子成员及领导干部的教育提醒和管理监督，认真贯彻执行廉洁自律《准则》和纪律处分《条例》，坚持高标准，守住纪律“底线”。深化作风建设，持之以恒纠正“四风”，结合“三严三实”专题教育重点整治基层干部损害群众利益问题。大力开展行政监察和审计监督，完善政府预算编制体系和财政监督检查机制。深入推进惩防体系建设和“权力入笼”工程，强化关键部门和重点岗位权力运行的监督制约，防控廉政风险。四是务实施政。大力实施干部素质提升工程，强化落实意识和担当精神，建立健全行政问责体系，严肃治理不作为、慢作为、乱作为和“中梗阻”问题，惩处失职渎职。加强政府决策落实情况的跟踪督查和绩效评估，做到责任有主体、实施有监督、结果有考核、优劣有奖惩，确保政令畅通，切实提升政府执行力。

　　各位代表！回顾过去，成就令人鼓舞；展望未来，蓝图催人奋进。让我们在省委、省政府和市委的正确领导下，开拓进取、奋力拼搏，为建设国家绿色转型发展示范区而努力奋斗！
